# Liquid Mixing on Falling Films: Marker-Free, Molecule-Sensitive 3D Mapping Using Raman Imaging

**DOI:** 10.3390/s23135846

**Published:** 2023-06-23

**Authors:** Marcel Nachtmann, Daniel Feger, Felix Wühler, Matthias Rädle, Stephan Scholl

**Affiliations:** 1 Center for Mass Spectrometery and Optical Spectroscopy, Hochschule Mannheim University of Applied Sciences, 68163 Mannheim, Germany; m.nachtmann@hs-mannheim.de (M.N.); d.feger@hs-mannheim.de (D.F.); f.wuehler@hs-mannheim.de (F.W.); 2 Institute for Chemical and Thermal Process Engineering, Technische Universität Braunschweig, 38106 Brunswick, Germany; s.scholl@tu-braunschweig.de

**Keywords:** falling film, Raman spectroscopy, non-contact measurement, flow characteristics, marker free, molecule sensitive, mixing

## Abstract

Following up on a proof of concept, this publication presents a new method for mixing mapping on falling liquid films. On falling liquid films, different surfaces, plain or structured, are common. Regarding mixing of different components, the surface has a significant effect on its capabilities and performance. The presented approach combines marker-free and molecule-sensitive measurements with cross-section mapping to emphasize the mixing capabilities of different surfaces. As an example of the mixing capabilities on falling films, the mixing of sodium sulfate with tap water is presented, followed by a comparison between a plain surface and a pillow plate. The method relies upon point-by-point Raman imaging with a custom-built high-working-distance, low-depth-of-focus probe. To compensate for the long-time measurements, the continuous plant is in its steady state, which means the local mixing state is constant, and the differences are based on the liquids’ position on the falling film, not on time. Starting with two separate streams, the mixing progresses by falling down the surface. In conclusion, Raman imaging is capable of monitoring mixing without any film disturbance and provides detailed information on liquid flow in falling films.

## 1. Introduction

Plant design requires different assumptions regarding liquid behavior during separation. These assumptions are necessary due to the lack of process and molecule data. A few well-described compounds provide the basis for derivation to other molecules. Caused by the lack of reliable data, suboptimal plant operation is possible. In many cases, the separation effort is overshot to avoid insufficient compound separation. From an energy point of view, fluid films are the most energy-intensive sub-task in large chemical plants. These separation columns require exorbitant amounts of energy, mostly for heating, but also for pumping processes. Usually, separation columns pass on their final or intermediate products obtained from the reactants to the next process stage [1,2]. Over many years of optimization, considerable progress has been made in the field of fluid handling in these separation processes. Still, the realization of technical aggregates and plants has not reached the point of no remaining improvement. In order to enhance this development, new process control tools are required. Process control allows close monitoring and alteration of the assumptions made [3]. These must not alter the films during the measurements. The goal therefore is a non-contact measuring method to provide information, which might be film thickness, mixing capabilities or heat transfer, on the current state of the process stages. Analyzing this information and providing it for process control and further process optimization is the superior goal. Related separation processes and separation aggregates, a non-contact measurement technique with high spatial resolution and molecule selectivity is required to solve process engineering challenges locally and supported with as many measurement points as possible in 2D or 3D images. 

In the context of the research presented, the proposed measurement technique is Raman imaging. It has disadvantages but also immense advantages. The disadvantages lie in the Raman effect itself, with extremely low excitation probabilities of the irradiated molecules and high excitation sources needed. The main advantage is that Raman spectroscopy as well as Raman imaging measurements are non-contact. Compared to near-infrared spectroscopy, Raman uses co-axial probes and does not require transmitted light, while being as molecule selective as mid-infrared spectroscopy. This allows us to reduce the amount of assumptions needed and to verify those made. Therefore, this publication proposes to complement the previously established methods, in which flow resistances influence the films or scanning operations obtaining summary information via near-infrared absorptions or fluorescence detection [4,5]. Raman scanning from a large working distance has not yet been established in the world of process engineering. The difficulties detecting such small effect with an association to strong lasers and highly sensitive sensors as well as the nearly unknown type of spectroscopy for process engineering led to an overseen and underestimated measurement technique. In laboratory use, however, Raman imaging receives increasing attention [5]. With broadly available, bright and stabilized laser sources as well as the development of new detection arrays with an increased sensitivity and improved signal–noise ratio, Raman spectrometry and imaging is trending in laboratory use and is slowly heading toward process engineering. Adapted to falling films, a newly developed scanning head system allows movement in all three dimensions without disturbing the films themselves, since the light focus is non-contact.

In this work, mixing processes are highlighted as an example unit to demonstrate the capabilities of Raman imaging in fluid films. Raman spectroscopy does not require any fluid labeling, and thus, any contamination, change in physical properties, triggering of reactions, catalytic effects and others are prevented.

### 1.1. State of the Art

Highlighting the state of the art, several different measurement approaches are available. The authors’ previous publication presents a summary on different Raman imaging approaches [6]. In the following section, the focus will be on falling films and the available approaches to allow a more complete view on the state of the art. 

#### 1.1.1. Local Film Thickness

Starting with the local film thickness, many approaches, intrusive and non-contact, are available. A detailed summary is available in a dissertation by F. Al-Sibais [7]. Summarizing his findings, film thickness measurements started in 1910 and 1918 by Hopf on trickle films, using micrometer screws quantity measurements [8]. The idea of micrometer screws and quantity measurements for measuring film thickness remained throughout different publications and over the course of 50 years [9,10,11,12]. 

Using optical procedures, fluorescence and near-infrared absorption spectroscopy or imaging are the dominant effects for determination of film thickness [4,13]. The fluorescence approach provides access to the local film thickness if any fluorescent material is available or added to the liquids, thus altering the composition of the liquid stream. Near-infrared imaging uses the absorption of the irradiated light and therefore does not alter the liquid streams, which is comparable to Raman imaging.

Concluding film thickness measurements, detection is possible using electrical conductivity. For these measurements, two electrodes with alternating current are placed on both sides of the film. The change in amplitude correlates with the film thickness [14].

#### 1.1.2. Concentration Measurements on Trickle and Falling Films

For concentration measurements on trickle and falling films, only 2D approaches or microscopic 3D approaches are available. Such 2D approaches as ultraviolet-visible (UV/VIS), fluorescence and near-infrared imaging provide local, molecule-sensitive and non-contact concentrations. Every 2D measurement point is a mean value over the local film thickness [4,15].

Lou et al. realized a transmission measurement set-up by adding UV light sources behind the film and the detection unit in front of the liquid film. The published study measures the removal of antibiotics from wastewater obtaining 2D local concentration information [15]. 

Tourvieille et al. published a method on mass transfer on micro-structured falling films using fluorescence-based confocal microscopy. The authors aimed to visualize lab-scale throughputs [16]. Despite the different measurement technologies, fluorescence and Raman, and the targeted environment, lab and industrial scale, the results of the methods are similar. For liquid compounds with an intrinsic fluorescence, no interruption nor alteration of the liquid streams occurs. Tracing different compounds with distinguishable fluorescence signals during mixing or chemical reactions, the mass transfer becomes visible. The main difference is in the aim of the methods. The confocal fluorescence microscopy has strict limitations regarding its measurement window. In contrast, a Raman imaging set-up can be adapted to larger falling films.

In conclusion, concentration measurements with a full 3D approach for industrial scale falling films are not available in the available literature. With Raman imaging, a new understanding of falling films and the concentration distribution is possible, due to its molecule-sensitive effect combined with non-contact measurements. In contrast to fluorescence measurements, a broader range of compounds is available with Raman imaging without altering the liquid stream. As a byproduct of these measurements comes the determination of local film thicknesses. For information on film thickness only, faster approaches are available, such as near-infrared imaging.

## 2. Materials and Methods

For a better and broader understanding, the following section starts with the basic theory of the relevant areas. Following up with the measurement set-up including the experimental plant and concluding the section with the design of experiments.

### 2.1. Trickle Film

Trickle films with different slopes are common in the chemical industry. Most relevant applications in the chemical industry are evaporation or condensation in so-called tube condensers or falling film evaporators. Falling films themselves are trickle films with a 90-degree slope and are commonly used for the evaporation of thermally unstable products, due to their high-heat exchanger surface compared to the low liquid volume [17]. Mixing or chemical reactions on falling films are also possible, but less common. As the focus is on mixing, this publication will not cover evaporation and heat transfer theory in this paragraph.

For the characterization of the present falling film, the minimum wetting density $\frac{\dot{V}}{B}$ is the most important characteristic number, as it defines the minimum volume flow $\dot{V}$ for a stable film for a given width B. In the literature, minimum wetting density under ideal conditions is between 0.5 and 1.5 $\frac{m^{3}}{mh}$ [17,18,19,20,21].

Another important characteristic number in falling films is the Reynolds number. The Reynolds number describes the turbulence in the present falling film. For Reynolds numbers below *Re* < 6, the falling film is laminar–smooth. Above *Re* > 300, the film is laminar–wavy. Above *Re* = 400, big and irregular waves can occur [17]. The Reynolds number can be calculated using the minimum wetting density $\frac{\dot{V}}{B}$ and the kinematic viscosity ν or the mass flow $\dot{M}$ with the dynamic viscosity *η* [17,18]. The correlation is given in Formula (1):

(1) $$Re=\frac{\dot{M}}{B\cdot \eta }=\frac{\frac{\dot{V}}{B}}{\nu }$$

Using the Reynolds number, *Re*, the kinematic viscosity ν as well as the acceleration of gravity *g*, the film thickness δ can be determined using Formula (2) [18]:

(2) $$\delta =({\frac{{3\nu }^{2}}{g})}^{\frac{1}{3}}\cdot {Re}^{\frac{1}{3}}$$

As minimum wetting density, Reynolds number and film thickness are interdependent, the experimental determination of at least one number is mandatory. For this publication, the minimum wetting density was determined experimentally. The minimum wetting density, as well as the resulting Reynolds number and film thickness, are specific for the individual plant.

### 2.2. Raman Imaging

Raman spectroscopy in general, as well as Raman imaging rely on the basic Raman effect. C.V. Raman discovered this effect and first published in 1928 [22]. The Raman effect can be sub-classified to the Stokes and Anti-Stokes scattering. When exciting a molecule with a monochromatic light source, the molecule shifts from its ground (i) or excited state (f) to a virtual energy level r or r’. Thus, not being stable, the molecule emits a photon and drops down to a stable energy level. This process can result in three different 2-photon-scatterings. Besides Raman scattering, an inelastic scattering, the most likely scattering is the Rayleigh scattering, an elastic scattering; thus, the molecule returns to its state prior to excitation. The frequency of the emitted photon hv_0_ remains identical to the excitation source. For Stokes scattering, the molecule is in its ground state i and is excited to a virtual energy level r, but it stays in an excited state f after the photon emission. Due to the energy remaining in the molecule, the frequency is reduced by v_r_, compared to the excitation source, and the wavelength increases. v_r_ is called the Raman shift and is specific for every molecule or functional group and relative to the excitation source. Regarding the Anti-Stokes scattering, the molecule has to be in an excited state f before being excited to the virtual energy level r’. After photon emission h (v_0_ + v_r_) the molecule returns to its ground state i. As the molecule releases energy, the frequency increases by v_r_, and the emitted scattering has a lower wavelength. In the presented work, proper optical filters remove the Anti-Stokes scattering as well as the Rayleigh scattering to allow only Stokes scattering observation and analysis [5,22,23,24,25,26,27,28,29,30].

The intensity of the Raman shift is bound to the molecules change polarization change (delta *a*/delta *r*), the intensity of the excitation source I_0_, the excitation frequency v by the power of 4, as well as the number n of molecules excited. Formula (3) provides the mathematical correlation [23].

(3) $$I\propto \nu^{4}\cdot I_{0}\cdot n\cdot \frac{\delta_{a}}{\delta_{r}}$$

### 2.3. Laminar and Turbulent Mixing

There are two different kinds of mixing operations, namely, purely laminar and purely turbulent mixing, as well as a combination of both.

Referring to turbulence, the fluid is in a state of motion with fluctuations of its velocity not only in time but also in all space directions. Due to its fluctuations of velocity, complex layering as well as vortices, sheets, ejections and sweeps can happen. Turbulence and strictly turbulent flow are linked to very high Reynolds numbers. In strictly turbulent flow, the viscous forces are negligible, and the inertial forces dominate via the flow, due to the high-velocity fluctuations. As turbulent flow is time dependent, a steady state is not achievable [31].

**Figure 1:**
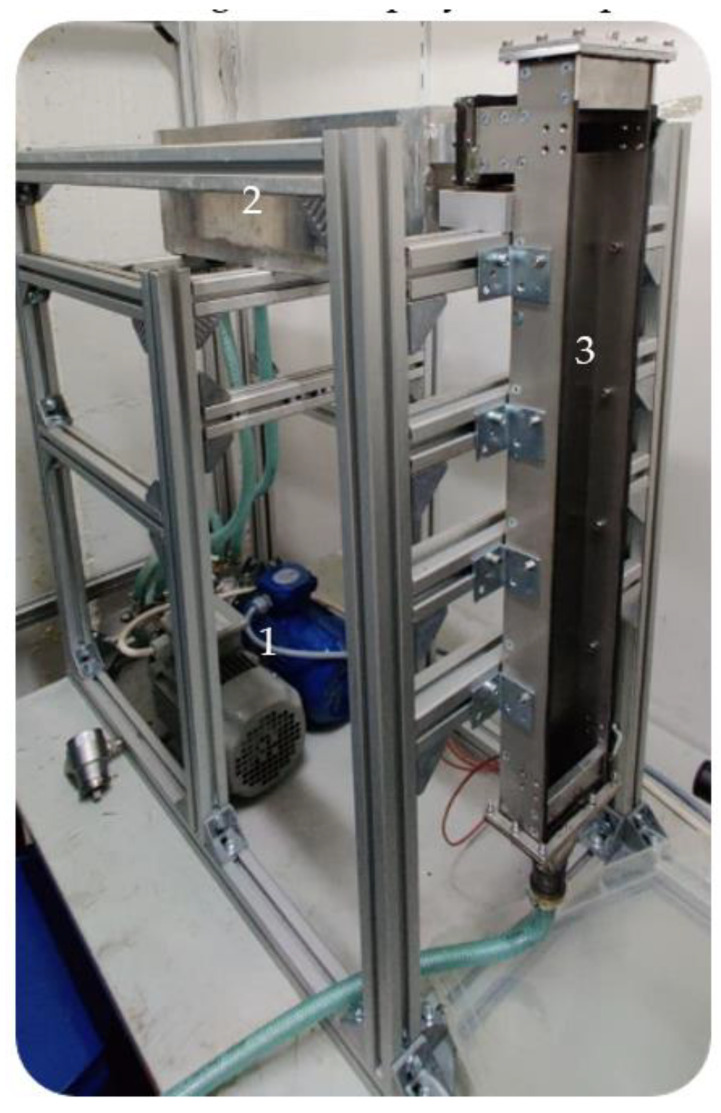
Plain surface experimental plant used for the mixing experiments. The measurement probe with the motorized linear stage are removed for better visibility.

Very low Reynolds numbers are an indicator for a strictly laminar flow. Steady laminar flows with a 2D flow mix due to diffusion, and compared to turbulent flow, the mixing performance is poor, Germany (1) maintaining flow from a reservoir to an overflow tank (2). Frequency converters (Siemens AG/Munich, Germany) allow the operator to fine-tuning the flow rate. The overflow tank (2) consists of two chambers and feeds the falling film (3). The tank mounts directly to the film. The falling film is prepared to fit different surfaces. In this publication, a plain surface and a pillow plate are installed. A disposal tank gathers the outflow. Figure 1 displays the experimental plant and Table 1 its individual dimensions.

**Figure 2:**
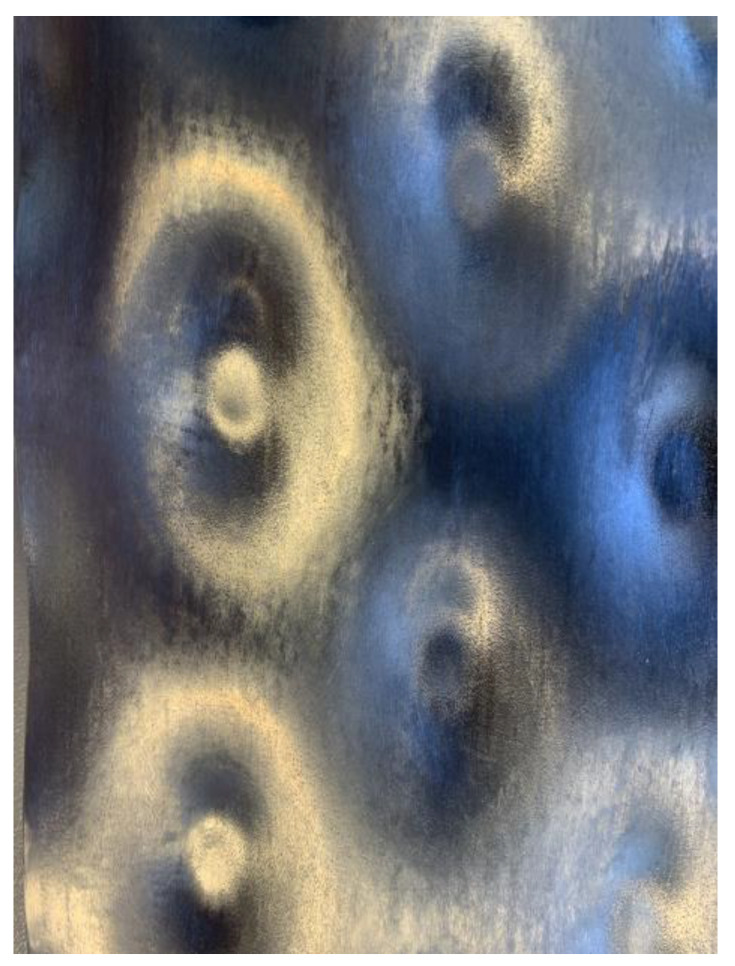
Close-up view of the 3D-printed pillow plate after polishing treatment.

The overflow tank is made from aluminum, the falling film itself, as well as the plain surface plate, are made out of 304 stainless steel. Sandblasting and polishing the plain surface ensure a constant surface finish. The pillow plate is a 3D-scanned reproduction of the described and characterized pillow plate by Piper et al. [32]. To fit the experimental plant, the 3D-scanned surface was modeled on an auxiliary construction and then 3D printed with 316 L stainless steel. Meeting the surface finish of the plain surface, the surface was carefully reworked. Figure 2 shows a close-up view of the pillow plate after its rework. 

To realize 3D measurements during mixing, a Raman probe is in front of the falling film and maneuvered with a motorized linear stage with three individual movable axes. A direct connection of the linear stage and the rack of the falling film prevents slipping. The minimum step size of the linear stage is 10 µm, and the reproduction accuracy is 5 µm. A LabVIEW (National Instruments Corp./Austin, TX, USA) program controls the motorized linear stage, while also connecting and synchronizing it with a Raman spectrometer (tec5 AG/Steinbach, Germany). The Raman probe is a self-built co-axial probe using 1-inch optics. This probe was carefully optimized for measurements at the experimental plant aiming for a tradeoff between small focal point, small depth of focus but also with a sufficient working distance to not disturb the film externally. The excitation source is an external laser source with single-mode fiber coupling. Table 2 lists the optical parameters.

The experimental plant is also described in a previous publication and has been altered to fit the new experiments. To rule out any sample alteration during measurements due to the high power laser, the proof of concept measurements use a closed circuit concept. The measurement duration was above six hours. No sample alteration could be measured over time [6].

### 2.4. Design of Experiments

**Figure 3:**
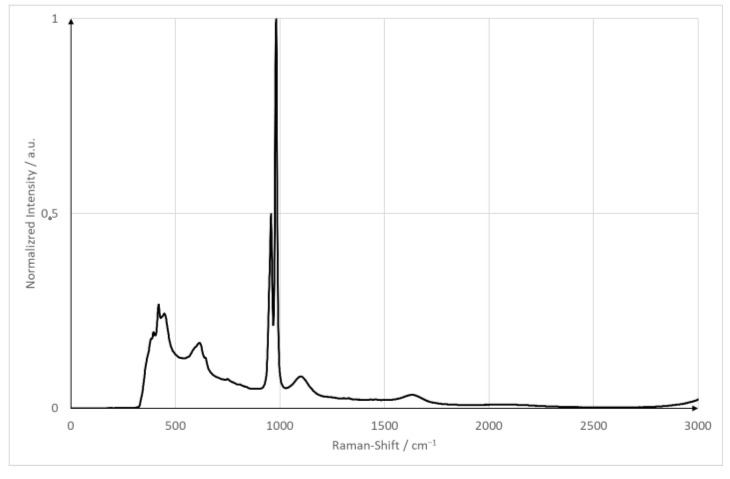
Raman spectrum of sodium sulfate hydrate.

The goal of the experiments is to compare a plain surface with a pillow plate regarding its mixing capabilities. For both surfaces, the flow is as laminar as possible while ensuring a stable film. The minimum wetting density limits the minimum Reynolds number. The Reynolds number is calculated using $\frac{\dot{V}}{B}$, the minimum wetting density and the kinematic viscosity. The kinematic viscosity is specific for every liquid. The minimum wetting density specifies the lowest Reynolds number possible. Below the minimum wetting density, no stable film is possible. For the plain surface, the Reynolds number is Re = 250; the pillow plate has a Reynolds number of Re = 360. Both films, therefore, are strictly laminar. The minimum wetting density in those cases are 0.9 $\frac{m^{3}}{mh}$ (plain surface) and 1.3 $\frac{m^{3}}{mh}$ (pillow plate). As pump parameters, both pumps were set to the same pumping capacity of 45 $\frac{L}{h}$ (plain surface) and 66.5 $\frac{L}{h}$ (pillow plate). As a reference, substance sodium sulfate is used. It has a strong Raman signal with an isolated peak at 950 cm^−1^, and it is neither toxic nor flammable. Figure 3 shows the spectrum of sodium sulfate measured with the equipment used in this publication [6]. The sodium sulfate concentration was set to 1 $\frac{Mol}{L}$. This concentration is identical compared to the proof-of-concept measurements. These parameters ensured a good signal-to-noise ratio and a good solubility in tap water at room temperature (20 °C/68 °F/293.15 K) without any solid particles remaining. The spectrometer parameters were adapted from the proof of concept and are five seconds with an accumulation of two [6]. Table 3 summarizes the plant and optical parameters.

Pre-measurements at several, individual coordinates on both surfaces specify the parameters for the actual measurements. In Table 4 summarizes the traversing parameters for the measurement slides. For the plain surface, the parameters could be limited due to the absence of any flow disruptors. Five measurement slides, from top of the falling film in equal steps over the maximum traversing path of the motorized linear unit, were acquired. The pillow plate with its structured surface is assumed to have better mixing. Taking this into account as well as the goal to enlighten the mixing behavior in one deepening, four slides are set from the top of the first pillow to its bottom. For a better comparison, a fifth slide is also at the end of the maximum traversing path. The parameters for each plain surface slide add up to 1919 measurement points over slightly above six hours and the pillow plate slides 1403 points over nearly five hours.

## 3. Results and Discussion

Starting with a short summary of the already published proof of concept [6], following up with the plain surface mixing and concluding with the pillow plate mixing.

**Figure 4:**
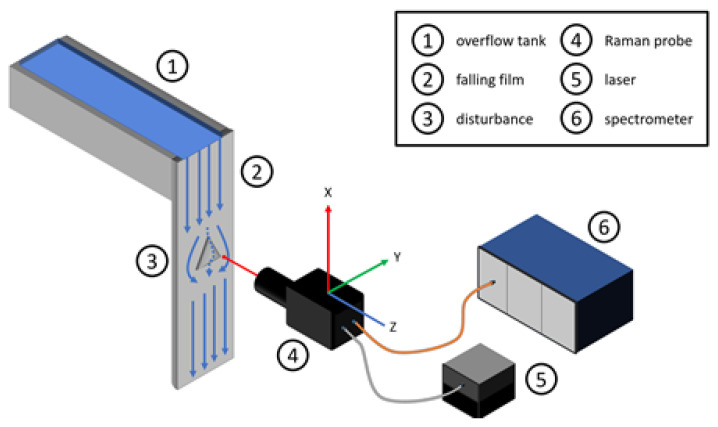
Schematic drawing of the experimental plant.

All slides share the same basic layout. Figure 4 displays a schematic layout of the experimental plant, containing the overflow tank (1), falling film (2), the plain surface with the pyramid attached (3), the Raman probe (4) as well as the laser source (5) and the spectrometer (6). Next to the probe, the schematic layout highlights the axes nomenclature for a better orientation, carrying these to the slide axes labeling. The abscissa depicts the linear stages’ y-axis, starting from zero on the left side of the falling film. Figuratively speaking, the y-axis is from left to right on the falling film, as well as in the resulting slides. On the ordinate, the z-values are plotted, starting from the respective backplates’ surface moving to thin air. The x-axis is from top to bottom and constant for each slide. The pseudo coloring indicates the measured normalized intensity. Blue or zero represent water and air, red to green or above zero indicates sodium sulfate concentration. The dominant peak at around 950 cm^−1^ represents the sodium sulfate concentration (see Figure 3). For further information on the calculation and data analysis, please refer to Section 3.1 or the previous publication [6]. Using the same probe layout with the identical optical components during all measurements, the focal point of 5.06 µm as well as the depth of focus of 75 µm remains constant and is visible in all measurement slides. The depth of focus and the focal point cause a blurring of the measurement signal, as a part of the excitation source is already in the sodium sulfate while the main focal point measures the water stream, or vice versa. During results description as well as the discussion in the following chapters, the resulting blur is already corrected.

### 3.1. Proof of Concept

Starting with a proof of concept, the experimental set-up was tested and validated. Following paragraph summarizes the already published for a better overview [6].

In a first step, pre-measurements determines the set-up performance on sodium sulfate detection in tap water on a falling film and eradicates possible experimental plant design flaws. For this cause, the plain surface was used, but with an artificially added pyramid shape in the middle of the surface. The pyramid is an example for the influence of flow disruptors on the flow behavior. In contrast to the measurements on mixing sodium sulfate in tap water with tap water, both chambers were filled with sodium sulfate in tap water with a concentration of 1 Mol sodium sulfate per liter tap water [6].

These measurements validate not only the plant itself, but also plant parameters, such as minimum wetting density, steady state of the film and general spectroscopic availability. For the plain surface, the minimum wetting density of 0.9 $\frac{m^{3}}{mh}$ − 1 $\frac{m^{3}}{mh}$ was determined and used in this publication. After ensuring a stable film and the steady state, the measurement set-up was optimized and validated again. The goal was to measure sodium sulfate in tap water on a falling film experimental plant. The Raman probe should have a small focal point, low depth of focus, as well as sufficient working distance. As these are competing effects, the goal is a trade-off with a sweet spot between the relevant parameters. The final Raman probe uses one-inch optics with a working distance of 16 mm, a focal point below 40 µm and a depth of focus of 75 µm. The Raman spectrometers (Multispec Raman spectrometer by tec5 AG/Steinbach, Germany) as well as the excitation source (MatchBox 785 nm single mode by Integrated Optics/Vilnius, Lithuania) were identical to the set-up in the present publication [6].

**Figure 5:**
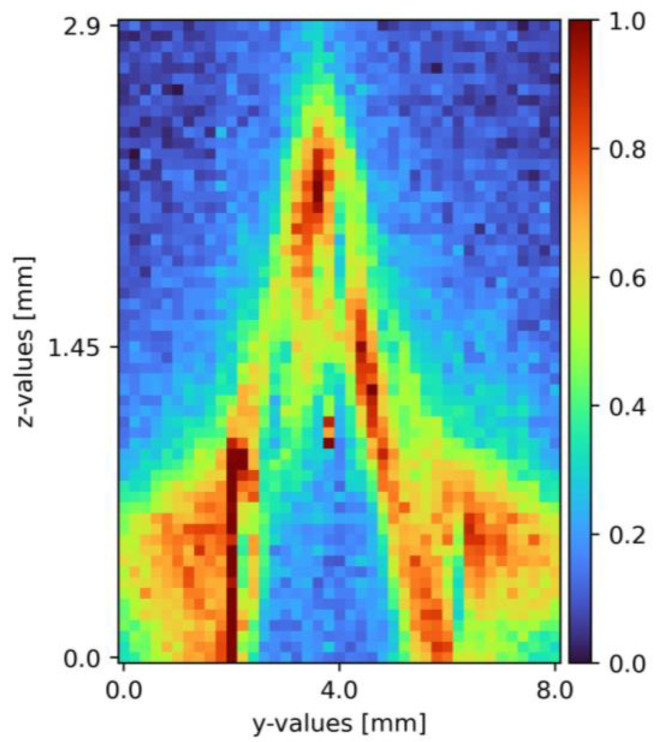
Cross section of sodium sulfate in tap water on a pyramid-shaped flow disruptor flowing down a plain surface falling film [6]. The liquid flows through the figure; z = 0 resembles the surface, z = 2.9 thin air. The false color scale represents the sodium sulfate concentration (see Section 3).

After the set-up validation, the actual proof of concept followed. The integration time was set to five seconds with an accumulation of two and 130 mW excitation power. During pretests, these settings arose as a sweet spot between measurement time and signal-to-noise ratio. Using these presets, measurements took place, using one mole per liter of sodium sulfate diluted in tap water. The x-axis was constant and aimed for the top of the pyramid; the y-axis segment was 8 mm wide with the top of the pyramid at 4 mm. The z-axis segment was 3.8 mm. A Python 3 tool (Python Software Foundation/Wilmington, NC, USA) analyzes the received data. First, the spectral range was limited, and zero values were due to the software, cosmic spikes, as well as data without any relevant information removed. In the following steps, an offset correction to the level of the lowest intensity value and a Savitzky–Golay filter was applied. Finally, yet importantly, a pseudo-coloring diagram with normalized pseudo-coloring visualizes the measurement points. Figure 5 shows one of the resulting cross-section slides as a pseudo-coloring diagram. The same data analysis methods are applied for the mixing measurements as well [6,33,34,35].

The proof of concept was provided, with the pyramid clearly visible in the cross-section (see Figure 5), with a sodium sulfate film covering its surface. To ensure the plant reached a steady state, measurement repetition showed with minor to none variation of the resulting cross-section pseudo-coloring diagrams. The plant validation was re-performed with the pillow plate as the surface. Solving challenges with the reflective surface, the data evaluation improved, and the cross-sections optimized in the progress. A more detailed description with additional measured pseudo-coloring diagrams is found in the previous publication [6].

### 3.2. Mixing on Plain Surface

**Figure 6:**
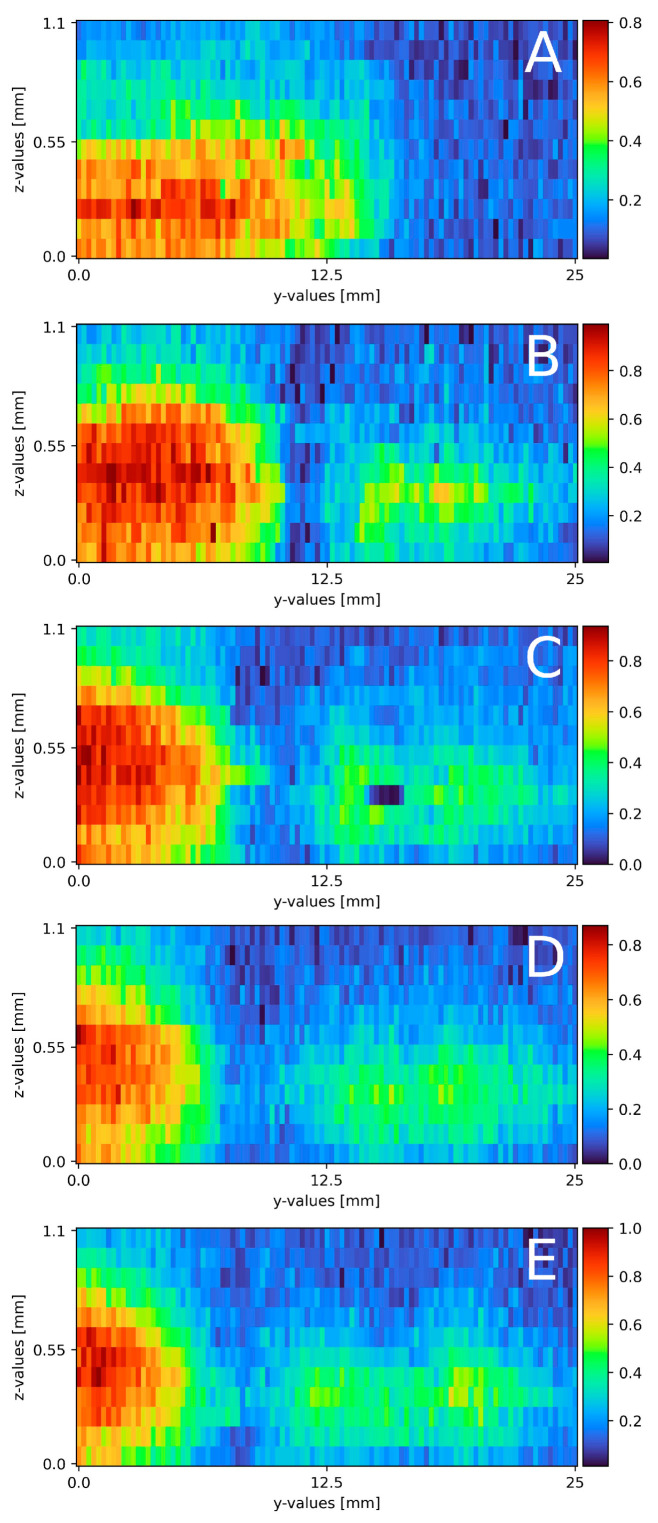
Results of the mixing process at different x-axis sections on a plain surface, (**A**) measured directly at the top of the falling film, x = 0. (**B**) x = 35 mm; (**C**) x = 70 mm; (**D**) x = 105 mm; (**E**) x = 145 mm. The liquid flows through the figure; z = 0 resembles the surface; z = 1.1 thin air. The false color scale represents the sodium sulfate concentration (see Section 3).

With a slightly altered experimental plant, compared to the proof of concept, mixing on a plain surface with laminar flow was measured. Figure 6 displays the results of all five cross-section slides. Starting from the top, the first cross-section is located right at the beginning of the falling film at x = 0 (a). A mixing area of about 3.5 to 4 mm separates the sodium sulfate and water phase. The sodium sulfate stream on the left side has a width of 10 mm and a width of 0.45 mm. At 35 mm down the falling film (b), the cross-section reveals a different concentration map with a highly concentrated sodium sulfate stream on the left side with a width of around 10 mm and a thickness of 0.45 mm. The mixing area is 1.5 to 2 mm wide. On the right side, an isolated partially mixed sodium sulfate stream becomes visible. The stream is 6 to 7 mm wide and 0.2 mm thick with a maximum concentration of 0.5 Mol/L. At 70 mm (c), the sodium sulfate phase is about 8 mm wide, 0.4 mm thick and has a mixing area of 2 to 2.5 mm to the water phase. The isolated sodium sulfate stream with 0.5 Mol/L starts mixing and widening up to 10 mm width. The depth remains constant. This trend continues for the slide at 105 mm (d), where the pure sodium sulfate stream flows to the left border of the measurement window with a decreased width of 6 mm and decreases to a thickness of 0.35 mm. The mixing area itself stays constant with a width of 2.5 to 3 mm. The isolated stream continues to widen to 12 mm at its maximum. The concentration drops to 0.4 Mol/L over the whole stream. In the last cross-section slide (e), the sodium sulfate phase is close to the left edge of the measurement window and is only visible for 4.5 mm. The thickness remains at 0.35 mm. The partially mixed area is around 4.5 mm wide. The isolated steam is about 13 mm wide and nearly connected to the main sodium sulfate stream with a concentration of 0.4 Mol/L. 

Compared to the theoretical film thickness of δ = 0.43 mm (see formula (2)), the measured film thickness of the sodium sulfate compartment matches the theoretical film thickness at the beginning of the mixture. During the mixing process, or in this case, moving down the film, the sodium sulfate stream thins out to 0.35 mm. This might be due to mixing, not only on the direct contact surface between both streams, but also on the top of the sodium sulfate stream, due to superposition of the water stream over the sodium sulfate stream. According to the theory, laminar streams do not or only partly mix while streaming in a pipe, tube or on a surface (see Section 2.3). Focusing on the sodium sulfate stream on the left, the theoretical course is also present in the measurements, as only a small part of the contact surface actually is involved in the mixing process, with its core being untouched. The stream moving to one side of the measurement window is due to slightly different volume flows of the rotary vane pumps. Even when both pumps and frequency converters are identical, a slight variation in both streams is present. With a variation in the frequency, the direction changes as well as the velocity. During mixing, an isolated sodium sulfate stream forms in the middle to right side. The area occupied by said stream expands, and the local concentration slightly reduces during the process. Compared to the main sodium stream, partial mixing is observed. As the isolated stream is not present during the first measurement, the mixing area, seen in Figure 6A, detaches from the main stream as the mixing areas decrease to Figure 6B and increase from 6b- e again. 

### 3.3. Mixing on Pillow Plate

**Figure 7:**
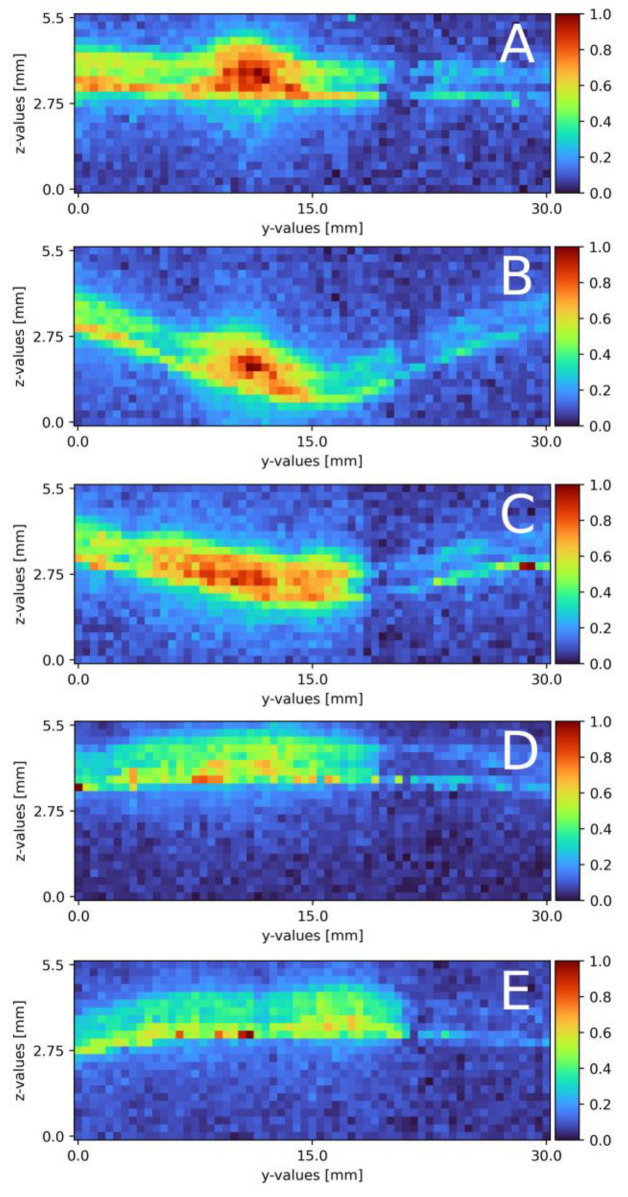
Results of the mixing process at different x-axis sections on a pillow plate surface. (**A**) measured at the top of the falling film directly before the first pillow, x = 0. (**B**) x = 10 mm; (**C**) x = 20 mm; (**D**) x = 30 mm; (**E**) x = 80 mm. The liquid flows through the figure; z = 0 resembles the surface, z = 2.8 thin air. The false color scale represents the sodium sulfate concentration (see Section 3).

Following the mixing on a plain falling film, the surface was swapped for a pillow plate. Section 2.4 describes the pillow plate in combination with the experimental plant. Compared to the plain surface mixing measurements, the volume flow needed some adjustment and the measurement windows increased. Table 3 and Table 4 list the exact parameters. Figure 7A–E displays the six cross-section slides. Starting from the top (Figure 7A), a distinct sodium sulfate stream in the middle of the measurement window is visible. The stream is 4 mm wide and 1 mm thick. Next to the stream, partially mixed areas are visible, from 0 to 9 mm on the y-axis a mixed area with a thickness of 1.2 mm and from 13 to 17 mm with a thickness of 1 mm. After 10 mm (Figure 7B), the cross-section is located in the first deepening of the pillow plate. The whole pillow structure is visible, due to a small (0.1 mm-thick) mixed layer directly on the surface across the whole width. The sodium sulfate stream remains in the identical place as in (Figure 7A). The width remains at 4 mm, as well as its thickness at 0.8 mm. The sodium sulfate stream mixes with the surrounding water, and therefore, its intensity reduces. The mixed areas, right and left from the sodium stream, remain constant. Moving further down to x = 20 mm (Figure 7C), the cross-section is on the end of the first deepening. The pillow profile is clearly visible over the whole slide, due to mixing areas on the left and right side of the cross-section slide. On the right side, from 17 to 30 mm, a thin layer of sodium sulfate of 0.1 mm is detected with a veil of 0.8 mm thickness. From 3 to 17 mm, the sulfate mixes to a normalized intensity of 0.5 to 0.6 $\frac{Mol}{L}$. The thickness of 1 mm remains constant. From 0 to 3 mm, a slightly mixed stream is visible at a thickness of 0.8 mm. Directly after the first pillow plates’ deepening (Figure 7D), the distinct sodium sulfate stream, compared to (Figure 7A,B) further reduces its intensity and a broad evenly mixed area is detectable. The mixed area ranges from 0 to 17 mm with a thickness of 1.2 mm. The normalized intensity ranges from 0.3 to 0.6. From 17 to 30 mm, the thin layer is visible again. The last cross-section slide is recorded at x = 80 mm (Figure 7E) and is similar to x = 40 mm (Figure 7D). The sodium sulfate mixture is visible from 0 to 20 mm with a thickness of 1.2 mm. The cross-section is located on the edge after a deepening. The surface itself is visible, due to the thin layer of sodium sulfate across the whole measurement window. This seems to thin out.

Compared to the theoretical film thickness on plain surfaces with the given Reynolds number of δ = 0.48 mm, the measured film thickness is increased by 0.5 to 0.7 mm. With a structured surface, a difference to the theory was expected. Regarding the sodium sulfate stream, the thickness remains constant over the whole measurement range. With the boundary condition of water being not detectable, the stream might seem thinner in some spots; compare this with Figure 7A on the left side of the sodium sulfate stream. In these areas, mixing already took place, or only water is present. In contrast to mixing on a plain surface, there is no strict separation of both stream to its respective side of the measurement window. In Figure 7A, a compact sodium sulfate stream is visible and mixes over the course of the measurement window. The uneven surface of the pillow plate might cause this. As discussed in following publication [32], two metal sheets are pressed together in the center of the deepening and the borders are welded together. Air is pressurized between the two sheets to form the final structure of the pillow plate [32]. With this procedure in mind, no surface area, except the areas pressed together, are even. In Figure 7A, besides being near the top of the film, mixing is already advanced compared to the plain surface. Falling down the film, the detached stream mixes from Figure 7A–E with the surrounding water. 

## 4. Conclusions

In conclusion, 3D concentration profiles on plain and structured surfaces falling films are accessible using Raman imaging. The proof of concept measurement results show a distinct correlation between sodium sulfate concentration and Raman signal even on structures such as a pyramid. Using these datasets, local film thickness surrounding individual structures or on structured surfaces can be derived. The presented measurement results show that this 3D concentration information also provides access to the concentration distribution of selected chemicals in a multi-stream or -component falling film. Depending on the chemicals used and distributed, starting with separated streams or pre-mixed streams, the film thickness can stay available. In this publication, a mixing application serves as an example, but Raman imaging is also capable of tracing reaction progress or condensation and evaporation in or to a falling film. At the state of the art, this information is only partially available (see Section 1.1). The novelty of this approach is the availability of 3D information without tracer substances or external film disturbance.

Regarding the two surfaces, the mixing performance differs. Comparing the mixed areas, the performance of the pillow plate is significantly higher after the first deepening, even though the plain surface had a much longer mixing distance. Both streams are laminar–wavy with comparable Reynolds numbers and film thicknesses. The pillow plate with its uneven surface besides the obvious pillow structure allows a significantly higher intermixing. The deepenings also boost the mixing performance. After the first deepening, the main sodium sulfate stream mixes completely with the surrounding water on the right side and the partially mixed stream on the left side. Once reaching a concentration of 0.4 to 0.6 $\frac{Mol}{L}$, the mixing performance reduces, but it still outperforms the plain surface. On the other hand, the plain surface had an individual stream detached from the main stream, which also distributed over the course of the available traversing path. The main stream remains nearly separated.

With the gathered results in mind, the Raman imaging approach can boost the understanding of liquid concentration distribution and therefore the performance of the targeted application. For mixing, as presented here, the mixing performance can be shown. For reaction tracing, the reaction progress as well as the product yield can be visualized. For evaporation or condensation, the enrichment or depletion of a target substance in a falling film can be detected. The main downside of the proposed method is the long measurement times. For complex matrices, the additional information of a Raman spectrometer is key, but matrices with only a few components with isolated peaks, a photometric approach might be favorable to drastically reduce measurement times. Another upside of a photometric approach is the possible excitation power reduction to meet explosion hazard regulations.

## Figures and Tables

**Table 1:** Dimensions and parameters of the experimental plant components.

Plant Component	Parameter	Value
Falling film surface	x	600 mm
	y	100 mm
	Minimum wetting density	$0.9\;\frac{m^{3}}{mh}$$(\mathrm{plain})-1.3\;\frac{m^{3}}{mh}$(pillow plate)
	Calculated film thickness	0.43 mm
Average pillow size	x	35 mm
	y	35 mm
	z	3 mm
Pump	Type	rotary vane
	Maximum flowrate (each)	150 L/h
	Maximum power outlet (each)	550 W

**Table 2:** Parameters of the optical components used in this publication.

Optical Component	Parameter	Value
Raman spectrometer	Wavelength	785 nm
	Optical range	320–3200 cm^−1^
	Optical resolution	5 cm^−1^
Laser source	Wavelength	785 nm
	Power	130 mW
	Optical coupling	Single-mode coupling
Raman probe	Focal point	5.06 µm
	Depth of focus	75 µm
	Working distance	16 mm
	Type	Co-axial

**Table 3:** Plant and optical parameters.

System	Parameter	Value
Optics	Integration time	5 s
	Accumulation	2
Liquid	Sodium sulfate	1 Mol/L
	Water	50 L
	Flow rate (each)	45 L/h (plain) 66.5 L/h (pillow plate)
	Temperature	20 °C

**Table 4:** Traversing parameters for the different 2D slides.

Surface	Axis	Distance	Step Size
plain	X	140 mm	35 mm
	Y	25 mm	0.25 mm
	Z	1.8 mm	0.1 mm
Pillow plate	X	40 mm	10 mm
	Y	35 mm	0.5 mm
	Z	5.5 mm	0.25 mm

## Data Availability

Not applicable.
